# Evaluation of CNS Depressant Activity of Different Plant parts of *Nyctanthes arbortristis* Linn.

**DOI:** 10.4103/0250-474X.49129

**Published:** 2008

**Authors:** Sanjita Das, D. Sasmal, S. P. Basu

**Affiliations:** Department of Pharmaceutical Sciences, Birla Institute of Technology, Mesra, Ranchi-835 215, India

**Keywords:** *Nyctanthes arbortristis* L., behavioral study, CNS depressant activity, histamine, serotonin

## Abstract

The present study was carried out with the water-soluble portion of the ethanol extracts of flowers, barks, seeds and leaves of *Nyctanthes arbortristis* Linn. to confirm their CNS depressant activity. The ethanol extracts of the plant parts were obtained by soxhlet extraction. After performing the gross behavioral study, the CNS depressant activity was evaluated by observing the prolongation of sleeping time induced by pentobarbital sodium in mice. Attempts have been made to explore the possible mechanism behind this activity by determining their effect on brain monoamine neurotransmitters like dopamine and serotonin. The gross behavioral study showed that ethanol extracts of the leaves, flowers and seeds possess significant CNS depressant activity. The leaves, flowers, seeds and barks (600 mg/kg) showed significant and dose-dependent prolongation of onset and duration of sleep and so found to cause decrease dopamine and increase serotonin level. From which it can be concluded that the CNS depressant activity of the ethanol extracts of seeds, leaves and flowers may be due to the decrease in dopamine and increase in serotonin level.

*Nyctanthes arbortristis* Linn. (Family Oleaceae), commonly known as *Harsingar* or Night Jasmine, is a common wild hardy large shrub or small tree^1^,^2^. *Nyctanthes arbortristis* L. is used by the rural people of Orissa in India to cure various ailments along with its use in Ayurveda, Sidha and Unani systems of medicines. It's claimed traditional uses have been proved on scientific basis using *in vitro* and *in vivo* experiments^3^–^7^. It has been established that its leaves possess hypnotic and tranquillizing activities^3^,^4^ and its flowers possess sedative activity^5^. The present study is aimed at the CNS depressant activity of the ethanol extracts of different parts (flowers, barks, seeds and leaves) of *Nyctanthes arbortristis* L. along with an attempt explore the responsible mechanism.

The flowers, barks, seeds and leaves of *Nyctanthes arbortristis* L. were collected from the garden of BIT, Mesra, Ranchi and forests of Orissa. The herbarium of the plant (CNH/I-I (20)/2005-Tech-II/254) was authenticated as *Nyctanthes arbortristis* L. from Botanical Survey of India, Kolkata. After drying properly, the leaves, barks and seeds were powdered coarsely and then were extracted successively with petroleum ether, chloroform and ethanol (90%)^6^,^7^, whereas its fresh flowers with ethanol (50%)^7^. The ethanol extracts were evaporated to dryness, having yield values 14%, 12.5%, 26.5% and 13% w/w, respectively. The water-soluble portions of the extracts were subjected to the pharmacological screening.

Adult male swiss mice weighing between 20-30 g, obtained from the animal house of BIT, Mesra, Ranchi, were used for this investigation. The Institutional Animals Ethics Committee (Registration No. 62/02/ac/CPCSEA) approved the experiments. Up and down or staircase method was followed for the estimation of acute toxicity of the water-soluble portion of the ethanol extracts of different parts of *Nyctanthes arbortristis* L. The dose was increased from 400 mg/kg to 2.0 g/kg, through intraperitoneal route of administration^8^,^9^.

The gross behavioral study was performed 30 min after the administration of the extracts to get maximum information about the effect of the extracts on the central nervous system of mice^9^. The control group of animals was only treated with pentobarbital sodium (45 mg/kg). Each extracts and the reference compound were injected 30 min before pentobarbital sodium administration. The time taken for the loss of righting reflex was noted in all cases. The onset of sleep was recorded by noting the time of loss of righting reflex of mice and duration of sleep by noting time difference between loss of righting reflex and recovery time^10^.

The monoamine neurotransmitters in brain were estimated following the method described by Shellenderger *et al*.^11^ The animals were sacrificed half an hour after the administration of the extracts by cervical dislocation. After chilling the head of mice in chilling mixture of ice and CaCl_2_, their brains were taken out and weighed^11^,^12^.

For the estimation of dopamine, brains were homogenized with dry *n*-butanol at 0°. Clear supernatant solutions were extracted with 0.1 M phosphate buffer. To the phosphate buffer extract 4% EDTA, Iodine solution, alkaline sulfite and 5N acetic acid were added and then heated. After cooling, the intensities of fluorescence were determined by the help of photofluorometer^11^,^12^.

For the estimation of serotonin, brains were homogenized with 0.1N HCl at 0° and then centrifuged. To the clear supernatant solutions, 10% zinc sulfate and 1N NaOH were added and then centrifuged. To a quartz cuvette containing 2N HCl the supernatant fluid was added and its intensity of the fluorescence was determined by the photofluorometer^11^,^12^. The results were expressed in mean±standard error of mean. The results were subjected to statistical analysis, using ANOVA to determine the significance of the tested activity and p<0.001 were considered to be significant^10^.

Toxicity studies revealed that the maximum tolerable dose for the water-soluble portion of the ethanol extracts was more than 2.0 g/kg body weights. From which, doses of 200, 400 and 600 mg/kg were selected for the evaluation of CNS depressant activity of the extracts. The gross behavioral study on mice of the extracts showed significant CNS depressant activity and some muscle relaxant activity. The leaves, flowers and seeds showed significant (p<0.001) and dose-related prolongation of the onset and duration of sleep, which are well comparable to that of the standard drug chlorpromazine (Table 1). It has been observed that the leaves possess highest CNS depressant activity.

**TABLE 1:** PROLONGATION OF PENTOBARBITAL-INDUCED SLEEPING TIME BY THE ETHANOL EXTRACTS OF DIFFERENT PARTS OF *NYCTANTHES ARBORTRISTIS* LINN.

Treatment	Dose (mg/kg)	Onset of Sleep (min)	Duration of Sleep (min)
Pentobarbitone	45	7.75±0.63	56.25±1.04
Chlorpromazine	3	4.75±0.48*	128.25±1.89*
NAF	200	5.75±0.25	81.00±1.87*
	400	5.50±0.29	89.50±2.06*
	600	5.00±0.205*	101.75±2.135*
NAB	200	7.75±0.79	70.25±2.625
	400	7.25±0.855	75.25±1.03*
	600	8.75±0.48	84.75±1.88*
NAS	200	6.00±0.41	83.50±1.89*
	400	4.25±0.48*	84.25±2.17*
	600	4.25±0.25*	100.5±3.07*
NAL	200	3.75±0.25*	86.75±2.84*
	400	3.75±0.48*	89.00±1.58*
	600	3.50±0.29*	106.25±0.95*

Values are expressed in Mean±SEM, n = 6

* p<0.001. NAF, NAB, NAS and NAL represent the ethanol extracts of the flowers, barks, seeds and leaves of *Nyctanthes arbortristis* respectively.

The changes in dopamine and 5-hydroxytryptamine (5-HT) levels, expressed as percentage increase or decrease from their respective control values, are presented in Table 2. The ethanol extracts of the leaves, seeds and flowers of *Nyctanthes arbortristis* L. were found to cause decrease in dopamine and increase in 5-HT level significantly in a dose-dependent manner. Its barks were found to cause these changes only at its higher dose of 600 mg/kg. Decrease in dopamine level may lead to the development of catalepsy and depression in the brain^13^. This can be well correlated with the antipsychotic activity of the extracts.

**TABLE 2:** EFFECT OF THE WATER-SOLUBLE FRACTION OF THE ETHANOL EXTRACTS OF *NYCTANTHES ARBORTRISTIS* ON MONOAMINE NEUROTRANSMITTERS IN THE BRAIN OF MICE

Treatment	Dose (mg/kg)	Dopamine		Serotonin	
			
		Level in brain tissue	Percentage decrease	Level in brain tissue	Percentage decrease
Control					
(Water for injection)	–	0.433±0.016	—	0.343±0.01	—
Chlorpromazine	3	0.205±0.01*	56.66	0.693±0.053	102.04
NAF	200	0.328±0.01*	24.25	0.423±0.013*	23.32
	400	0.283±0.021*	34.25	0.488±0.009*	42.27
	600	0.243±0.071*	44.0	0.635±0.01*	85.13
NAB	200	0.413±0.035	4.62	0.345±0.017	0.58
	400	0.400±0.016	7.62	0.328±0.009	4.37
	600	0.33±0.014*	23.79	0.44±0.014*	28.28
NAS	200	0.358±0.005*	17.44	0.400±0.0.016	16.62
	400	0.295±0.018*	31.87	0.528±0.015*	53.94
	600	0.25±0.008*	42.26	0.588±0.008*	71.43
NAL	200	0.313±0.001*	27.83	0.448±0.005*	30.47
	400	0.273±0.065*	37.07	0.525±0.018*	53.06
	600	0.233±0.015*	46.19	0.638±0.012*	86.01

Values are expressed in Mean±SEM;

* p<0.001. NAF, NAB, NAS and NAL represent the water-soluble fraction of the ethanol extracts of the flowers, barks, seeds and leaves of *Nyctanthes arbortristis*, respectively.

Many antipsychotic agents also have some affinity for 5-HT receptors and drugs, which increase brain 5-HT and usually increase sleep^14^. This is evident in case of the extracts treatment where the 5-HT level is increased. This can be well correlated with the potentiation of pentobarbitone-induced sleeping time in mice by the extracts, which could be accounted to the increase in the concentration of 5-HT in the brain.

The overall study confirms that the CNS depressant action and the potentiation of pentobarbitone-induced sleeping time by the ethanol extracts of flowers, seeds, leaves and barks (600 mg/kg) of *Nyctanthes arbortristis* L. might be due to the decrease in dopamine and increase in the 5-hydroxytryptamine level in brain.
